# Infective Endocarditis Caused by Non-HACEK Gram-Negative Bacteria, a Registry-Based Comparative Study

**DOI:** 10.1093/ofid/ofaf085

**Published:** 2025-02-13

**Authors:** Jasmina Al Janabi, Mohammed El Noaimi, Torgny Sunnerhagen, Ulrika Snygg-Martin, Magnus Rasmussen

**Affiliations:** Division of Infection Medicine, Department of Clinical Sciences Lund, Lund University, Lund, Sweden; Division of Infection Medicine, Department of Clinical Sciences Lund, Lund University, Lund, Sweden; Division of Infection Medicine, Department of Clinical Sciences Lund, Lund University, Lund, Sweden; Department of Clinical Microbiology, Infection Control and Prevention, Office for Medial Services, Lund, Sweden; Department of Infectious Diseases, Institute of Biomedicine, Sahlgrenska Academy, University of Gothenburg, Gothenburg, Sweden; Department of Infectious Diseases, Sahlgrenska University Hospital, Gothenburg, Sweden; Division of Infection Medicine, Department of Clinical Sciences Lund, Lund University, Lund, Sweden; Department of Infectious Diseases, Skåne University Hospital, Lund, Sweden

**Keywords:** Duke-ISCVID, infective endocarditis, nHGNB, *Pseudomonas aeruginosa*, *Serratia marcescens*

## Abstract

**Background:**

Infective endocarditis (IE) caused by non-HACEK gram-negative bacteria (nHGNB) is uncommon. In the 2023 Duke-ISCVID diagnostic criteria, *Pseudomonas aeruginosa* and *Serratia marcescens* were added as “typical” pathogens. We examine the consequences of this addition, the risk of IE in bacteremia from nHGNB species, and the features of IE caused by nHGNB.

**Methods:**

nHGNB IE cases reported to the Swedish Registry of Infective Endocarditis (SRIE) between 2008 and 2023 were identified. Episodes of bacteremia caused by nHGNB during the same period in Region Skåne were used as controls. Characteristics of IE caused by nHGNB were compared with those of other pathogens reported to the SRIE.

**Results:**

One hundred fourteen episodes of nHGNB IE, of which 98 (87%) were definitive, were identified (1.5% of all cases). *Escherichia coli* was the most common cause (28%), followed by *Pseudomonas aeruginosa* (13%) and *Klebsiella* (9%). Applying the Duke-ISCVID criteria, none of the possible IE episodes caused by *P. aeruginosa or S. marcescens* were reclassified as definitive IE. Comparing the proportion of nHGNB species in episodes with IE with the proportion of nHGNB species in episodes with bacteremia (n = 33 213), *E. coli* was more common in bacteremia than in IE, whereas *P. aeruginosa* and *Serratia* were more common in IE. Patients with nHGNB IE frequently had underlying diseases, and mortality was higher than in streptococcal IE.

**Conclusions:**

Our findings indicate that *P. aeruginosa* and *Serratia* are more common in IE than in bacteremia but that that their status as “typical IE pathogens” in the Duke-ISCVID criteria did not improve the performance of the criteria.

Infective endocarditis (IE) is an infection of the endocardium or on intracardiac prosthetic material such as a prosthetic valve and/or a cardiovascular implantable electronic device (CIED) [1]. *Stapylococcus aureus* and alpha hemolytic streptococci, followed by enterococci and HACEK (*Haemophilus* other than *H. influenzae, Aggretibacter, Cardiobacterium, Eikinella,* and *Kingella*) are the most common bacteria that cause IE [2]. On rare occasions, non-HACEK gram-negative bacteria (nHGNB) cause IE [3], and the incidence of such infections has increased in recent years [4]. This rise has been suggested to be related to an elderly population, increased vascular catheterization, and intravascular prosthetic materials [2, 5]

The proportion of IE due to nHGNB is low and ranges from 1.8% to 3.9% in different studies. *Escherichia coli, Pseudomonas aeruginosa, Klebsiella* spp., and *Serratia marcescens* have been reported to be the most common causative species of nHGNB IE episodes [6, 7]. Different predisposing factors for nHGNB IE have been reported, and comorbidities such as diabetes, heart failure, active neoplasia, chronic kidney disease, and hemodialysis appear to be common [8]. The proportion of persons who inject drugs (PWID) was high among those with nHGNB IE in some of the studies, and in non-PWID predisposing factors such as implanted endovascular devices and central venous catheter were common [3, 8–11]. The episodes of IE caused by nHGNB were reported to be predominantly nosocomial rather than community acquired [3, 4, 10]. Clinical outcomes included a higher proportion of persons subjected to surgery and a higher risk of re-admission. Trends toward an increased risk of mortality compared with patients with IE caused by other pathogens, both in-hospital and at 1 year, have been seen in patients with IE caused by nHGNB [6, 8, 10].

Diagnosis of IE is based on the Duke criteria. These criteria are composed of imaging criteria and microbiological criteria as well as some minor criteria that include predisposition for IE and IE-related symptoms. The Duke criteria were first published in 1994 [12], they were modified in 2000 [13], and they were recently updated by the International Society for Cardiovascular Infectious Diseases (the Duke-ISCVID criteria) [14]. Among other changes, the Duke-ISCVID criteria added 2 species of nHGNB, *Pseudomanas aeruginosa* and *Serratia marcenscens*, as “typical” IE pathogens, but only in conjunction with intracardiac prosthetic material [14]. This change was based on data from a prospective cohort study conducted on hospitalized patients with monomicrobial bacteremia due to nHGNB and cardiac devices [15].

In this study, we investigate the species distribution of nHGNB IE in Sweden and compare that with the distribution of nHGNB among all-cause bacteremia in our region. We also aim to investigate whether the Duke-ISCVID criteria classified more episodes treated as IE caused by nHGNB as definite IE. Moreover, our aim was to explore the clinical characteristics of nHGNB IE and compare them with other common IE-causative genera.

## METHODS

### Episodes of IE

All the episodes of IE were gathered through the Swedish registry of Infective Endocarditis (SRIE), which is organized by the Swedish Society of Infectious Disease. It holds electronical records of patients treated for IE in Sweden since 1995. In 2008, an internet-based reporting system was established, and the design of it was modified in 2018. All 30 departments of infectious diseases have been involved since the beginning. These departments are responsible for the treatment of IE in each region. Reported cases of IE caused by nHGNB, HACEK, *Stapylococcus aureus,* alpha-hemolytic streptococci, and enterococci between 2008 and 2023 were extracted and compared. No imputations were made when data were missing. Polymicrobial IE was regarded as IE caused by each of the pathogens, and thus the number of episodes is higher than the number of patients. Episodes of possible IE caused by nHGNB were reclassified with the Duke-ISCVID criteria where *P. aeruginosa* and *S. marcescens* were regarded as typical IE pathogens in conjunction with coronary implanted material, which was defined as either a pacemaker/ICD or a heart valve prothesis [14].

### Control Episodes of Bacteremia

Data regarding all episodes of bacteremia caused by nHGNB were received from the Department of Clinical Microbiology at Skåne University Hospital. Data included all episodes of bacteremia from 2008 to 2023 that were caused by gram-negative bacteria from the region of Skåne, which had a population of between 1 200 000 and 1 400 000 persons [16]. The episodes thus occurred during 21 million person-years. There were no data on clinical features of the patients, and some of them might have had IE. The population of Skåne is in many ways similar to that of all of Sweden [16].

### Statistics

The pairwise comparisons for categorical variables were made utilizing the Fisher exact test. As the continous variables were found to be non–normally distributed using the Shapiro-Wilk test, we applied the Mann-Whitney *U* test for comparisons of such variables. The chi-square test was performed in the analysis on the proportion of species in bacteremia and IE. Significance was defined as a *P* value <.05. GraphPad Prism, version 10.2.0, was used for statistical calculations.

## RESULTS

### IE Caused by Non-HACEK Gram-negative Bacteria

The SRIE at this time point held information on 7426 episodes of definite or possible IE according to the modified Duke criteria included between 2008 and 2023. nHGNB were identified as causative agents in 114 episodes, corresponding to 1.5%, of which 98 (87%) were reported as definitive IE. *Escherichia coli* was the most common causative species (n = 34). Twenty percent of the episodes were polymicrobial (n = 23). The complete list of IE caused by nHGNB is presented in Table 1.

**Table 1.** nHGNB in Episodes of Infective Endocarditis and Bacteremia

Non-HACEK Gram-Negative Bacteria	Episodes of IE, n = 114 (%)	Definitive IE, n = 99 (%)	Polymicrobial, n = 23 (%)	Episodes of Bacteremia, n = 29 147 (%)
*Escherichia coli*	34 (28)	27 (79)	4 (12)	18 141 (55)
*Pseudomonas aeruginosa*	16 (13)	15 (84)	2 (13)	1387 (4)
*Klebsiella pneumoniae*	8 (7)	6 (75)	0	3139 (9)
*Haemophilus influenzae*	7 (6)	5 (71)	0	301 (1)
*Enterobacter cloacae*	7 (6)	7 (100)	4 (57)	576 (2)
*Serratia marcescens*	6 (5)	3 (50)	1 (17)	383 (1)
*Stenotrophomonas maltophilia*	4 (3)	4 (100)	3 (75)	157 (0.4)
*Capnocytophaga canimorsus*	3 (2)	3 (100)	0	66 (0.2)
*Klebsiella oxytoca*	3 (2)	3 (100)	1 (33)	1331 (4)
*Proteus mirabilis*	3 (2)	3 (100)	0	1267 (4)
*Morganella morganii*	2 (2)	1 (50)	1 (0)	245 (0.7)
*Neiserria meningitidis*	2 (2)	1 (50)	0	52 (0.2)
*Pasturella multocida*	2 (2)	2 (100)	0	62 (0.2)
*Yersinia enterocolitica*	2 (2)	2 (100)	0	7 (0.02)
*Acinetobacter baumanii*	1 (1)	1 (100)	1 (100)	8 (0.02)
*Acinetobacter* spp.	1 (1)	1 (100)	1 (100)	156 (0.5)
*Acinetobacter ursingii*	1 (1)	1 (100)	0	11 (0.03)
*Achromobacter xyloxidans*	1 (1)	1 (100)	0	18 (0.05)
*Bacteroides fragilis*	1 (1)	1 (100)	0	918 (3)
*Bordetella holmesii*	1 (1)	0	0	0
*Bacteroides* spp.	1 (1)	0	0	0
*Campylobacter fetus*	1 (1)	1 (100)	0	0
*Citrobacter freundii*	1 (1)	1 (100)	1 (100)	305 (0.9)
*Citrobacter koseri*	1 (1)	1 (100)	0	246 (0.7)
*Moraxella* spp.	1 (1)	0	0	28 (0.08)
*Neiserria elongata*	1 (1)	1 (100)	0	4 (0.01)
*Neiserria* spp.	1 (1)	0	1 (100)	57 (0.2)
*Pantoea* spp.	1 (1)	1 (100)	0	16 (0.05)
*Pasturella dagmatis*	1 (1)	1 (100)	0	0
*Prevotella* spp.	1 (1)	1 (100)	0	32 (0.09)
*Proteus vulgaris*	1 (1)	1 (100)	0	82 (0.2)
*Pseudomonas* spp.	1 (1)	1 (100)	1 (100)	60 (0.2)
*Rhizobium* spp.	1 (1)	1 (100)	1 (100)	0
*Salmonella* spp.	1 (1)	0	0	3 (0)
*Sphingomonas paucimobilis*	1 (1)	1 (100)	1 (100)	60 (0.2)
*Veionella parvula*	1 (1)	1 (100)	0	29 (0.09)

Abbreviations: IE, infective endocarditis; nHGNB, non-HACEK gram-negative bacteria.

### Duke-ISCVID Criteria

Next we determined if the changes in the diagnosis of nHGNB IE introduced in the Duke-ISCVID criteria would change some of the episodes identified as “possible IE” by the modified Duke criteria to “definite IE” by the new criteria. Among the 16 *P. aeruginosa–*caused episodes, 8 (50%) were in patients with cardiac devices, and there was only 1 episode classified as possible IE. This episode of possible *P. aeruginosa* IE did not fulfill definitive criteria by applying the new criteria either. Among the 4 episodes of *S. marcescens* IE, 3 episodes were in patients with cardiac devices and 3 were classified as possible IE with the modified Duke criteria. None of the *S. marcescens* IE episodes were reclassified to “definitive IE” using to the Duke-ISCVID criteria (details of the episodes eligible for reclassification are given in Supplementary Table 1).

If the Duke-ISCVID criteria would have included all nHGNB as typical pathogens in patients with intracardiac prosthesis material, an additional 3 episodes with possible IE would have been eligible for reclassification. In 2 of these 3 episodes, such a hypothetical change would have led to reclassification from possible IE to definite IE. See Supplementary Table 2 for details.

### Proportion of nHGNB in IE in Relation to the Proportion of nHGNB in Bacteremia

**Figure 1.**
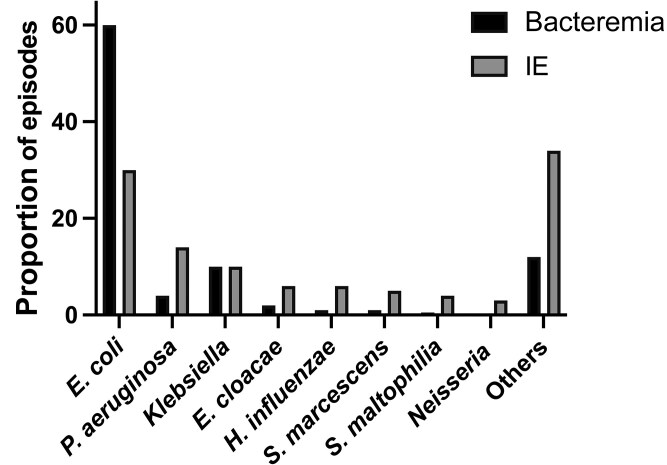
The proportions of episodes with bacteremia (in dark gray) and IE (in light gray) with nHGNB caused by the respective species are shown. Other species are shown in Table 1 and Supplementary Table 1. The difference in distribution was significant using the chi-square test (*P* < .001). Abbreviation: IE, infective endocarditis.

The proportions of episodes of IE (114 episodes reported to SRIE) caused by the most common species and genera were compared with the proportions of the same genera and species in a control population consisting of all 33 213 episodes of bacteremia with nHGNB during the same period in a population-based cohort in Southern Sweden. The results are depicted in Figure 1 and detailed in Table 1. *E. coli* was the only species where the proportion among patients with bacteremia was higher than the proportion among patients with IE. In contrast, the proportions of *P. aeruginosa, Haemophilus influenzae*, *Enterobacter cloacae, Serratia marcenscens, Stenotrophomonas maltophilia,* and *Neisseria* were higher in IE than in bacteremia. The proportions of the different species and genera in the IE group were significantly different from the proportions of the same bacteria in bacteremia (*P* < .0001 with chi^2^ test).

### Comparison of IE Caused by nHGNB and IE Caused by Other Pathogens

Statistical comparisons of features of IE episodes caused by the 2 most common species of nHGNB, *E. coli* (n = 34) and *P. aeruginosa* (n = 16), were performed. The other species were too uncommon to allow for statistical comparisons. Higher proportions of patients with previous IE and right-sided IE were observed in episodes caused by *P. aeruginosa* than in those caused by *E. coli* (*P* = .002 and *P* = .03 respectively). The clinical characteristics can be seen in Table 2.

**Table 2.** Characteristics of Infective Endocarditis Caused by Different Species in the nHGNB Group

Characteristics	Non-HACEK Gram-Negatives, n = 121	*E. coli,* n = 34	*P. aeruginosa,* n = 16	*K. pneumoniae* & *Oxytoca,* n = 11	*H. influenzae,* n = 7	*E. cloacae,* n = 7	*S. marcescens,* n = 6	*S. maltophilia,* n = 4	*Neiserria* spp., n = 4	Other, n = 35
Age, median, y	69 (26–93)^a^	75 (51–93)	63.5 (29–88)	59 (26–81)	68 (38–87)	45 (26–79)	43 (26–64)	39 (33–57)	62 (55–89)	70 (26–88)
Male gender, No. (%)	67 (55)	15 (45)	12 (75)	7 (64)	4 (57)	4 (57)	6 (100)	0	3 (75)	18 (51)
Underlying disease, No. (%)										
Diabetes	33 (27)	9 (26)	5 (31)	5 (45)	0	2 (29)	1 (17)	1 (25)	0	10 (29)
Cancer	18 (15)	8 (24)	2 (13)	5 (45)	0	0	0	0	1 (25)	5 (18)
IVDU	24 (20)	1 (3)	3 (19)	6 (55)	1 (14)	5 (71)	3 (50)	4 (100)	1 (25)	4 (11)
Underlying heart disease, No. (%)										
Native valve disease	14 (12)	5 (15)	3 (19)	0	2 (29)	1 (14)	0	1 (25)	1 (25)	0
Prosthetic heart valve	26 (21)	11 (32)	4 (25)	1 (9)	1 (14)	1 (14)	1 (17)	0	0	7 (20)
Previous IE, No. (%)	19 (16)	0	5 (31)**	2 (18)	1 (14)	1 (14)	4 (67)	1 (25)	1 (25)	5 (14)
Pacemaker/ICD, No. (%)	17 (14)	5 (15)	4 (25)	2 (18)	0	1 (14)	2 (33)	0	1 (25)	4 (11)
Predisposing factors for IEb,^b^ No. (%)	68 (56)	18 (53)	9 (56)	5 (45)	3 (43)	7 (100)	5 (83)	4 (100)	2 (50)	17 (49)
Definite IE, No. (%)	99 (82)	27 (79)	15 (84)	9 (82)	5 (71)	7 (100)	3 (50)	4 (100)	2 (50)	86 (30)
Type of infection, No. (%)										
NVE, left	75 (62)	19 (56)	6 (38)	7 (64)	7 (100)	4 (57)	0	3 (75)	4 (100)	26 (74)
NVE, right	10 (8)	0	3 (19)*	2 (18)	0	2 (29)	3 (50)	0	0	1 (3)
PVE	26 (21)	10 (29)	4 (25)	2 (18)	0	1 (14)	2 (33)	1 (25)	0	7 (20)
CIED	10 (8)	5 (15)	3 (19)	0	0	0	1 (17)	0	0	1 (3)
Polymicrobial, No. (%)	23 (19)	4 (12)	2 (13)	1 (9)	…	4 (57)	1 (17)	3 (75)	1 (25)	23 (8)
Site of acquisition, No. (%)										
Community	96 (79)	26 (81)	10 (67)	11 (100)	6 (86)	7 (100)	6 (100)	4 (100)	4 (100)	71 (25)
Nosocomial	11 (9)	3 (9)	3 (20)	0	0	0	0	0	0	14 (5)
Health care associated	6 (5)	3 (9)	2 (13)	0	0	0	0	0	0	3 (1)
Time from disease										
onset to hospitalization, d	7 (0–200)	3 (0–60)	5 (0–108)	10 (0–28)	4 (1–14)	14 (1–200)	7 (1–7)*	5 (1–26)	12 (0–43)	6 (0–39)
Length of stay, d	36 (0–88)	39 (8–88)	43 (10–86)	39 (10–71)	31 (0–67)	33 (13–50)	38 (28–73)	36 (32–54)	23 (16–55)	36 (8–85)
Treatment length, d	30 (8–120)	34 (10–120)	42 (18–55)	33 (17–60)	34 (28–75)	28 (13–43)	30 (29–42)*	31 (14–51)	28 (17–32)	29 (8–58)*
Embolization, No. (%)	37 (31)	6 (18)	4 (25)	7 (64)	3 (43)	6 (86)	1 (17)	2 (50)	1 (25)	8 (23)
Surgery, No. (%)	54 (45)	14 (41)	9 (56)	6 (55)	1 (14)	4 (57)	1 (17)	4 (100)	2 (50)	14 (40)
In-hospital mortality, No. (%)	21 (17)	7 (21)	3 (19)	0	1 (14)	3 (43)	0	1 (25)	0	6 (17)

Abbreviations: CIED, cardiac implantable electronic device; ICD, implantable cardioverter defibrillator; IE, infective endocarditis; IVDU, intravenous drug use; nHGNB, non-HACEK gram-negative bacteria; NVE, native valve disease; PVE, prosthetic valve disease.

*P < .05; **P < .01; ***P < .001.

^a^Numbers in brackets for continuous variables indicate the range.

^b^Predisposing factors for IE were prosthetic valve, previous IE, IVDU, and native valve disease.

Next, the features of IE caused by nHGNB and IE caused by other common IE pathogens (*S. aureus,* alpha hemolytic streptococci, enterococci and HACEK) were compared. Table 3 summarizes the results. Underlying diseases such as diabetes and cancer were significantly more common in patients presenting with IE due to nHGNB than in IE caused by other bacteria. PWID constituted 20% of patients with nHGNB or *S. aureus* IE but were less common in IE caused by other pathogens. It was more common that patients with IE due to nHGNB had a history of previous IE. Patients with IE due to nHGNB also presented more commonly with left-sided native valve IE compared with *S. aureus* (*P* < .05) and enterococci (*P* < .01).

**Table 3.** Clinical Characteristics of the nHGNB Group Compared With HACEK, *S. aureus,* Alpha-streptococci, and Enterococci

Characteristics	Non-HACEK Gram-Negatives, n = 114	HACEK, n = 150	*S. aureus,* n = 2875	Alpha-streptococci, n = 2070	Enterococci, n = 804
Age, median, y	69 (32–89)^a^	65 (17–97)	68 (2–99)	71 (8–99)	75 (22–99)***
Male gender, No. (%)	67 (59)	116 (77)**	1778 (62)	1481 (72)**	610 (76)***
Underlying disease, No. (%)					
Diabetes	33 (29)	11 (7)***	569 (20)*	298 (14)***	158 (20)*
Cancer	18 (16)	4 (3)***	278 (10)*	216 (10)	132 (16)
IVDU	24 (21)	5 (3)***	614 (21)	74 (4)***	82 (10)**
Underlying heart disease, No. (%)					
Native valve disease	14 (12)	39 (26)**	333 (13)	600 (29)***	142 (18)
Prosthetic heart valve	26 (23)	53 (35)*	406 (14)*	575 (28)	309 (38)**
Previous IE, No. (%)	19 (17)	12 (8)*	243 (8)**	199 (10)*	117 (15)
Pacemaker/ICD, No. (%)	17 (15)	30 (20)	477 (17)	176 (9)*	163 (20)
Predisposing factors for IEb,^b^ No. (%)	68 (60)	84 (55)	1617 (56)	1193 (58)	551 (69)
Definite IE, No. (%)	98 (87)	101 (66)***	2334 (81)	1527 (74)**	628 (78)
Type of infection, No. (%)					
NVE, left	75 (66)	77 (51)	1501 (52)*	1294 (63)	395 (49)**
NVE, right	10 (9)	8 (6)	598 (21)**	60 (3)**	46 (6)
PVE	26 (23)	38 (28)	386 (13)**	556 (27)	276 (34)*
CIED	10 (9)	13 (9)	318 (11)	60 (3)**	77 (10)
Polymicrobial, No. (%)	23 (20)	13 (9)	…	…	…
Site of acquisition, No. (%)					
Community	96 (91)	139 (93)*	2254 (78)	1900 (92)**	642 (80)
Nosocomial	11 (10)	6 (4)	387 (13)	76 (4)**	83 (10)
Health care associated	6 (6)	2 (1)	99 (3)	25 (1)**	32 (4)
Time from disease					
onset to hospitalization, d	7 (0–200)	12 (1–317)**	2 (0–375)***	5 (0–773)	3 (0–389)***
Length of stay, d	36 (15–85)	30 (3–131)*	33 (0–175)	30 (0–157)***	39 (0–161)
Treatment length, d	30 (8–68)	32 (3–103)	30 (0–300)	29 (0–175)**	39 (1–999)*
Embolization, No. (%)	37 (32)	34 (23)	1018 (35)	401 (19)**	162 (20)**
Surgery, No. (%)	54 (47)	58 (39)	740 (26)***	503 (24)***	213 (26)***
In-hospital mortality, No. (%)	21 (18)	4 (3)***	412 (14)	114 (6)***	89 (11)*

Abbreviations: CIED, cardiac implantable electronic device; ICD, implantable cardioverter defibrillator; IE, infective endocarditis; IVDU, intravenous drug use; nHGNB, non-HACEK gram-negative bacteria; NVE, native valve disease; PVE, prosthetic valve disease.

*P < .05; **P < .01; ***P < .001.

^a^Numbers in brackets for continuous variables indicate the range.

^b^Predisposing factors for IE were prosthetic valve, previous IE, IVDU, and native valve disease.

There were multiple differences in the course of disease. Patients with IE due to nHGNB suffered from embolic events in one-third of the cases (32%), which is a similar proportion as in *S. aureus* IE patients (35%) but significantly more frequent compared with alpha streptococci and enterococci IE (19% and 20%, respectively; *P* < .01). Additionally, patients with nHGNB IE were more often subjected to heart valve surgery than those infected with *S. aureus*, alpha streptococci, and enterococci (47% of the patients compared with 26%, 24%, and 26%; *P* < .001). Mortality was also significantly higher in nHGNB IE (18%) compared with HACEK, alpha-streptococci, and enterococci (3%, 6%, and 11%, respectively).

## DISCUSSION

Our results are in concordance with previous reports about IE caused by nHGNB in terms of the proportion of total IE caused by nHGNB and the clinical features of IE caused by nHGNB. We did not identify any improvement of sensitivity from the change introduced in the 2023 Duke-ISCVID criteria indicating that *S. marcescens* and *P. aeruginosa* should be considered typical IE pathogens. The episodes reported as possible IE to the SRIE remained possible also with the new Duke-ISCVID criteria. However, due to the very few cases of possible IE caused by these pathogens reported to our registry, we cannot rule out that the changes may have increased the sensitivity slightly, especially in populations with a higher proportion of cardiac devices. Three episodes of possible IE caused by other nHGNB (*E. coli* and *Neisseria*) would have been reclassified from possible to definite IE if the Duke-ISCVID criteria had suggested that all nHGNB be regarded as “typical IE pathogens” in patients with intracardiac foreign material. The potential slight gain in sensistivity of including more bacteria as typical IE pathogens must be balanced against a risk of overdiagnosis, particularly in patients classified as having a possible IE. The design of this study does not allow an analysis of the potentially decreased specificity of the changes in the Duke-ISCVID criteria.

Similar to other studies, we found that the most frequent nHGNB pathogens in IE were *E. coli* and *P. aeruginosa* [6, 7]. However, unlike previous studies in the field, we compared the proportions of different nHGNB in patients with IE and in patients with bacteremia. A weakness in this comparison is that a few patients with bacteremia might have had IE. However, this comparison showed clearly that despite *E. coli* being numerically the most common cause of nHGNB IE, the risk for IE in *E. coli* bacteremia is lower than in bacteremia with other pathogens such as *S. marcescens*, *P. aeruginosa, H. influenzae, E. cloacae, S. maltophilia, and Neisseria* spp. Therefore, despite the fact that the addition of *S. marcescens* and *P. aeruginosa* as typical IE pathogens in the Duke-ISCVID criteria did not improve the performance of the criteria, we agree that *S. marcescens* and *P. aeruginosa* are more “typical IE pathogens” than, for example, *E. coli* and *Klebsiella.* Moreover, the Duke-ISCVID criteria especially pointed out the connection of *S. marcescens* and *P. aeruginosa* to prosthetic materials, and indeed a high proportion of patients with *P. aeruginosa* and *S. marcescens* IE had intracardiac prosthetic materials.

This study is hampered by the fact that the comparisons made between the proportion of bacteremia and IE episodes caused by different bacteria are not based on the same population. The SRIE holds all episodes of IE in the 30 different departments of infectious diseases, while the data regarding bacteremia and the proportion of all episodes of bacteremia are based only on numbers in 1 region. We believe, however, that the incidence and species distribution of nHGNB bacteremia are similar in our region and Sweden as a whole, making the comparison putatively valid. Our region has all types of hospitals, and very few patients are referred to other regions. Moreover, there is only 1 microbiology laboratory serving all hospitals and care facilities in the Skåne region, making the data on the bacteremia population-based.

We demonstrate that comorbidities were more prevalent in those with nHGNB IE as compared with those with IE caused by other pathogens. For example, the prevalence of diabetes and cancer was higher in the nHGNB-IE group than in IE caused by other pathogens. PWID were overrepresented in the nHGNB-IE group; injection drug use was as common as in the *S. aureus–*IE group. All these observations confirm earlier observations [3, 8, 11]. Our findings suggest that patients with nHGNB IE are more likely to undergo heart valve surgery and have a higher risk for a fatal outcome as compared with patients who suffer from IE caused by another pathogen. It is not clear if this is due to the higher burden of comorbidities of the patients with nHGNB IE or to the infection being more severe.

Strengths of our study include a large cohort of 114 episodes and a possibility to compare the species distribution with that of all-cause bacteremia. Another strength is that the episodes were collected from registries based on the entire population and not on tertiary centers reporting to central registries. Weaknesses include the retrospective design of the study, which limits the availability of data. Another weakness is the risk for misclassification of episodes in the SRIE. One scenario is that cases of nHGNB IE are missed in the clinic because these pathogens are not believed to cause IE and therefore not reported to the registry, causing a falsely low proportion of IE. Another scenario is that bacteremia with nHGNB is erroneously believed to be IE in patients with valvular lesions from, for example, previous IE, thus leading to false-positive IE episodes in the registry. Additionally, some bacteria reported may represent contamination or be from other concurrent infections such as intravenous line infections, especially in polymicrobial cases. It is also a weakness that the SRIE is not complete as reporting of cases is voluntary. However, the risk for missingness is likely not related to the pathogen causing IE, making the estimates of proportions reliable. Moreover, as also pointed out above, it is a weakness that nHGNB IE and nHGNB bacteremia are not studied in the same population.

In conclusion, IE caused by nHGNB is uncommon but affects persons with comorbidities and has a severe course of infection. Several nHGNB were overrepresented in IE patients in comparison with patients with bacteremia, whereas *E. coli* was underrepresented. The inclusion of *P. aeruginosa* and *S. marcescens* as typical IE pathogens in the Duke-ISCVID criteria may therefore be supported by the present findings, although this change did not improve the sensitivity of the criteria in our cohort.

## Supplementary Material

ofaf085_Supplementary_Data

## Supplementary Data

Supplementary materials are available at *Open Forum Infectious Diseases* online. Consisting of data provided by the authors to benefit the reader, the posted materials are not copyedited and are the sole responsibility of the authors, so questions or comments should be addressed to the corresponding author.
