# Supplementary material for: Infective Endocarditis Caused by Non-HACEK Gram-Negative Bacteria, a Registry-Based Comparative Study
Source: Open Forum Infect Dis. 2025 Feb 13;12(3):ofaf085. doi: 10.1093/ofid/ofaf085 (PMC11886782; doi:10.1093/ofid/ofaf085)
Supplement: ofaf085_Supplementary_Data [file ofaf085_supplementary_data.docx]

**Supplemental Table 1.** Reclassification of episodes with possible IE caused by P. aeruginosa and S. marcescens

| Episodes of possible IE caused by *P. aeruginosa* or *S. marcescens* | **Modified Duke** |  | **Duke-ISCVID**  Minor criteria |  | **Change of diagnosis?** |
| --- | --- | --- | --- | --- | --- |
| ***P. aeruginosa*** in three cultures. No vegetation on UCG^1^. Prosthetic valve. Previous IE. IVDU. Fever. | One major Two minor |  | One major  Two minor |  | Still possible IE |
| ***S. marcescens*** in two cultures. No vegetation on UCG. Previous IE. IVDU.  Fever. | No major  Three minor |  | One major  Two minor |  | Still possible IE |
| ***S. marcescens*** in 12 blood cultures. No findings on UCG. Pacemaker. No fever | One major  No minor |  | One major  One minor |  | Changed from reject to possible |
| ***S. marcescens*** in 4 out of 8 cultures. No findings on UCG. Prosthetic valve. IVDU. | No major  Two minor |  | One major One minor | . | Changed from reject to possible |

^1^Abbreviations used were UCG, ultrasound cardiography; IE, infective endocarditis; IVDU, intravenous drug use

**Supplemental Table 2.** Hypothetical reclassification of episodes with possible IE caused by nHGNB other than P. aeruginosa and S. marcescens

| Episodes of possible IE | **Modified Duke** |  | **Duke-ISCVID**  Minor criteria |  | **Change of diagnosis?** |
| --- | --- | --- | --- | --- | --- |
| ***E. coli*** in two cultures. No vegetation on UCG^1^. Pacemaker. Fever. | No major  Two minor |  | One major  Two minor |  | Change from reject to possible IE |
| ***E.coli*** in two cultures. Vegetation on UCG. Pacemaker.  Fever. | One major  Two minor |  | Two major  Two minor |  | Change from possible to definite IE |
| ***Neisseria*** species in two blood cultures. Vegetations on UCG. Pacemaker. Fever | One major  Two minor |  | Two major  Two minor |  | Change from possible to definite IE |

^1^Abbreviations used were UCG, ultrasound cardiography; IE, infective endocarditis; IVDU, intravenous drug use
